# Sporadic detection of vaccine-derived poliovirus type 2 using next-generation sequencing in Canadian wastewater in August of 2022

**DOI:** 10.1038/s41598-025-92912-x

**Published:** 2025-04-15

**Authors:** Grace E. Seo, Russell Mandes, Nathan D. Wright, Justin P. Hawkins, Anneliese Landgraff, Ravinder Lidder, Umar Mohammed, Chand S. Mangat, Anne-Sophie Michel, Judith Fafard, Darian Hole, Andrea D. Tyler, Marli Vlok, Elsie Grudeski, Tim F. Booth, Anna Majer

**Affiliations:** 1 Enterovirus and Enteric Viruses Laboratory, Viral Diseases, National Microbiology Laboratory, Public Health Agency of Canada, 1015 Arlington Street, Winnipeg, MB R3E 3R2 Canada; 2 Wastewater Surveillance Unit, National Microbiology Laboratory, Public Health Agency of Canada, Winnipeg, MB Canada; 3 Laboratoire de santé Publique du Québec, Institut national de santé publique du Québec, Ste-Anne-de-Bellevue, Québec Canada; 4 Computational and Operational Genomics, National Microbiology Laboratory, Public Health Agency of Canada, Winnipeg, MB Canada; 5 Department of Medical Microbiology and Infectious Diseases, Faculty of Health Sciences, College of Medicine, University of Manitoba, Winnipeg, MB Canada; 6 Ministère de la Santé et des services sociaux du Québec, Québec, Québec Canada

**Keywords:** Poliovirus, Vaccine-derived poliovirus type 2 (VDPV2), Wastewater surveillance, Next generation sequencing, Nanopore, Direct detection, VP1, Population screening, Whole genome amplification

## Abstract

In July 2022, an unvaccinated young adult living in Rockland County, New York became paralyzed due to a vaccine-derived poliovirus type 2 (VDPV2) infection that was internationally transmitted. Wastewater surveillance in New York State uncovered VDPV2 in neighboring counties, showing silent community spread. These communities have strong epidemiological links to some vaccine-hesitant communities in Canada, spurring the need to monitor these populations for potential poliovirus importation. At the time, Canada did not have an established poliovirus wastewater method. We initiated this study to apply molecular methods to detect poliovirus in these communities and establish poliovirus wastewater surveillance capabilities in Canada. We sequence confirmed one poliovirus detection on August 30th, 2022 using both viral isolation and direct detection methods. Subsequent retrospective and prospective sampling was initiated, resulting in another sequence confirmed detection of VDPV2 in an overlapping catchment area collected on August 27th, 2022. Both VDPV2 detections were genetically linked to the New York clinical case. No clinical cases of poliomyelitis were detected in Canada during this study. The sporadic detection of VDPV2 in Canadian wastewater supports a travel-related shedding event without community transmission. Furthermore, we show that a direct detection method is sensitive to sequence confirm poliovirus in wastewater samples.

**Supplementary Information:**

The online version contains supplementary material available at 10.1038/s41598-025-92912-x.

## **Introduction**

In Canada, the last case of poliomyelitis that was caused by an indigenous wild-type poliovirus occurred in 1977. In 1994, the American region (North and South America) was officially certified as poliovirus-free by the Regional Certification Commission for polio eradication^1^. To support Canada’s ongoing poliovirus-free status, the Canadian Acute Flaccid Paralysis Surveillance System (CAFPSS) was established in 1991 to monitor for acute flaccid paralysis, a major clinical presentation of poliomyelitis. Criteria for inclusion are patients who are < 15 years of age and who experience sudden onset of muscle weakness or paralysis^2^. However, this surveillance system does not detect silent poliovirus transmission within a population. The Global Polio Eradication Initiative (GPEI) recommends that countries such as Canada (*i.e.* having good overall poliovirus vaccine rates and long-term polio-free status) establish temporary environmental surveillance (ES) for poliovirus in response to an active outbreak or risk of poliovirus importation^3^.

In July of 2022, an unvaccinated young adult living in Rockland County, New York became paralyzed due to poliovirus infection^4,5^. Viral culture and genotyping confirmed the presence of vaccine-derived poliovirus type 2 (VDPV2). Sequence analysis determined that the VDPV2 detected in the New York clinical case shared common genetic changes observed in other VDPV2 sequences identified from ES sites in Israel and the UK, supporting international transmission^6–8^. Wastewater surveillance was established in Rockland and surrounding counties in response to the clinical case. The presence of VDPV2 was detected in Rockland and surrounding counties between April and October 2022, indicating ongoing and silent local transmission^5,9^. These developments posed a potential risk of poliovirus importation into Canada. The fact that poliovirus-positive communities in New York have strong epidemiological links to some communities in Canada that are vaccine-hesitant further increased the risk of poliovirus importation. As a result, Canada needed to enhance national poliovirus surveillance to include wastewater monitoring on an ad hoc basis^10^. This involved developing an operational wastewater surveillance capacity, including establishing laboratory methods for poliovirus detection and sequence confirmation from wastewater samples, as well as plans to strategically monitor select communities. At-risk communities that were monitored were based on their low vaccine coverage on official registries and frequent travel to and visitors from New York counties with active poliovirus transmission.

Environmental surveillance can inform public health of potential viral transmission prior to the detection of clinical cases^11,12^. Having an early-warning system can help trigger effective public health measures such as vaccine campaigns. In Israel, environmental surveillance for poliovirus uncovered the presence of prolonged silent transmission, which resulted in the emergence of poliovirus variants of public health concern^13^. Similarly, wastewater surveillance for poliovirus established in London, UK^7^ and recently in the USA as a response to the paralytic case detected in New York State^14^, helped reveal the extent of the local outbreak. Wastewater monitoring for poliovirus can therefore serve as an effective early-warning system and can provide impetus to rapidly initiate targeted vaccine campaigns in order to limit viral transmission and protect vulnerable populations from poliomyelitis.

The current gold standard for monitoring poliovirus in wastewater is to isolate poliovirus in culture followed by sequencing the VP1 region^15^. Specifically, ≥ 6 nucleotide differences within the entire 906 nucleotide VP1 region classify the poliovirus strain as vaccine-derived poliovirus type 2 (VDPV2)^16^. Reliable sequence data is therefore necessary to confirm the presence of poliovirus in complex environmental specimens and to distinguish circulating poliovirus of public health concern from the vaccine strain. Sequence information is also critical to link viral transmissions locally and internationally. Laboratory confirmation of poliovirus detection is the cornerstone to informing public health of the type of poliovirus identified and the extent of an outbreak. However, viral isolation takes considerable time (~ 2 weeks), requires dedicated infrastructure which includes extensive biocontainment requirements^17,18^, and can result in reporting delays due to challenges with logistics and resources^19^. Poliovirus genomes may also accrue mutations during viral culturing^20^ and low-fitness poliovirus variants that are common in complex specimens such as wastewater may become masked by dominant variants^21^. Furthermore, viral isolation by culture was shown to be less sensitive at detecting poliovirus than using molecular methods such as real-time PCR^22^. To address these challenges, methods for sequencing poliovirus genomes directly from wastewater are currently in development. One approach showing great promise for environmental surveillance applications uses nested PCR followed by Nanopore sequencing of the VP1 region. This approach was able to distinguish complex poliovirus mixtures from environmental samples^23^.

Our goal for this pilot study was to use molecular methods to detect poliovirus from wastewater that can be rapidly implemented to enhance surveillance in response to a poliovirus outbreak or event in Canada. This is the first study to detect and sequence confirm the presence of VDPV2 in Canadian wastewater. Sequence analysis linked Canadian VDPV2 to New York and international outbreaks. No clinical cases of poliomyelitis were detected in Canada during the time of this study. Lastly, our data further supports that a direct detection method can be sensitive enough to sequence confirm poliovirus from wastewater samples, including re-purposed COVID-19 surveillance specimens.

## **Results**

**Fig. 1:**
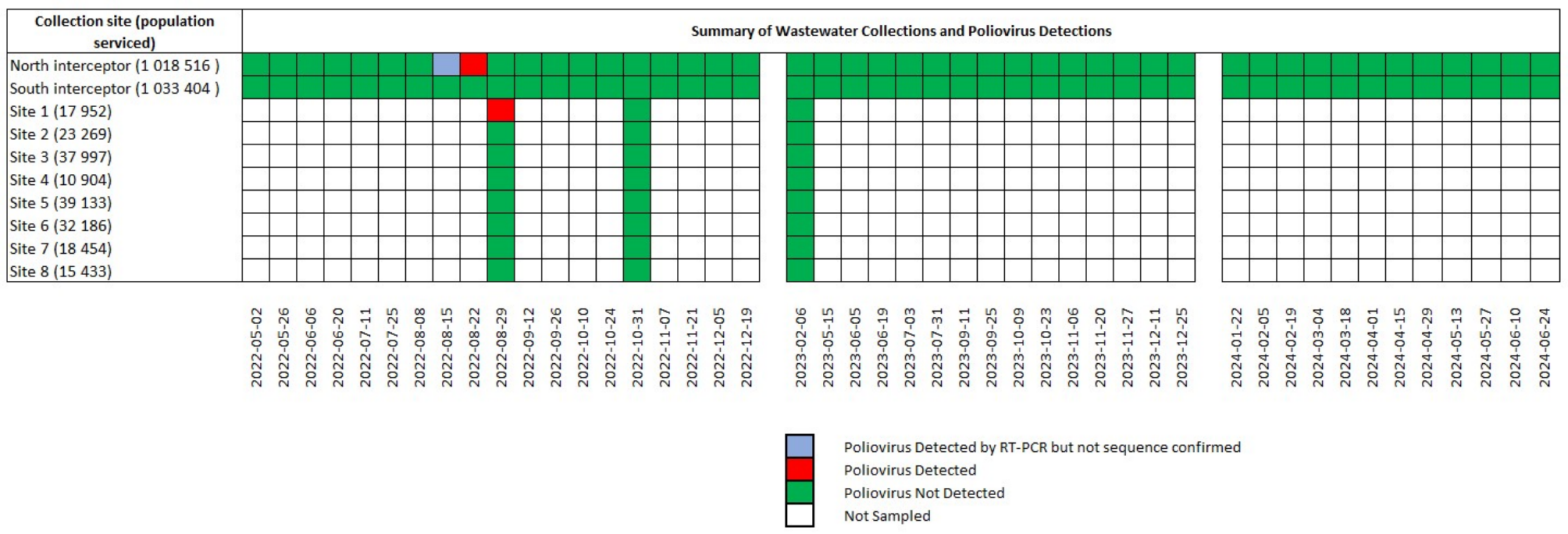
Summary of poliovirus detections in collected wastewater sampled between May 2022 and July 2024. Summary of the weeks when specimens were collected for each collection site. The dates represent the Monday of the weeks during which specimens were collected for poliovirus testing. The exact collection dates can be found in Supplemental Table S2. Samples for all targeted sites were specifically collected for poliovirus investigation while samples from the north and south interceptor pipes were re-purposed COVID-19 surveillance specimens. Estimated population serviced by each collection site is indicated. Poliovirus detections represent both real-time PCR (RT-PCR) poliovirus positive signals plus sequence confirmation.

**Fig. 2:**
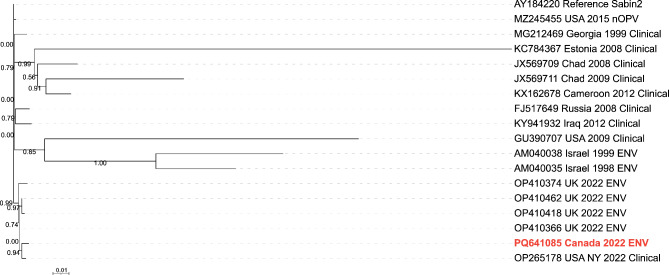
Maximum-likelihood phylogeny of the Poliovirus 2 capsid sequences. Nucleotide sequences of select poliovirus 2 VP1-VP4 coding region were obtained from the NCBI GenBank database. Red text highlights the poliovirus type 2 isolate sequence obtained from Canadian wastewater in 2022. The tree was rooted with a Sabin 2 reference. SH-like branch support values are indicated at the nodes and the maximum-likelihood scale bar indicates average residue substitution per site. ENV = environmental.

Wastewater samples were collected between May 2022 and June 2024 from Montréal’s main north and south interceptor pipes that direct wastewater to the city’s wastewater treatment plant plus up to 8 targeted sewage sites located within the city. All samples (*n* = 116) collected for this study were initially screened by Pan Enterovirus (PanEV) real-time PCR for non-polio enteroviruses (NPEVs) and Pan Poliovirus (PanPV) real-time PCR for poliovirus 1, 2 and 3 serotypes. The original PanEV and PanPV real-time assays designed for clinical application were unable to detect any enterovirus signal within our wastewater samples. However, by modifying the assays slightly we found that both PanEV and PanPV were able to detect enteroviruses and polioviruses, respectively (Supplemental Figure S1 and Table S1). We utilized the PanEV real-time PCR assay to determine the presence of NPEVs and therefore confirm the suitability of using the samples for further poliovirus testing as outlined in the GPEI environmental surveillance guidance documents^15,24^. Using the modified real-time PCR assays we found that all collection sites were positive for NPEVs > 95% of the time, confirming the suitability of using these samples for further poliovirus testing (Supplemental Table S2). Subsequent real-time PCR PanPV screening of these samples identified that one specimen collected on August 30th, 2022 from Site 1 screened positive for poliovirus by PanPV real-time PCR (Fig. 1 and Supplemental Table S2) and was referred for viral isolation in cell culture. Sanger sequencing of the VP1 region from the cultured viral isolate revealed the presence of 6 nucleotide substitutions when compared to the Sabin 2 VP1 reference sequence (AY184220.1), classifying this isolate (WW22-4017) as a VDPV2 (Supplemental Table S3)^16^. Phylogenetic analysis of VP1 sequences revealed that our isolate matched closely to several environmental VDPV2 isolates collected from London, UK as well as the New York clinical specimen (Supplemental Figure S2). Pair-wise analysis of the VP1 region revealed 99.4% identity with other environmental VDPV2 isolates sequenced from UK London sewage (OP410366.1) and 99.0% identity with the clinical isolate from New York State, USA-NY-22 (OP265178.1). In addition, our culture isolate shared 4 unique nucleotide substitutions within the VP1 region of the New York isolate, further supporting genetic linkage. Whole genome sequencing of our poliovirus isolate revealed a 99.5% identity between the environmental VDPV2 detected in UK London sewage (OP410366.1) as well as the clinical isolate from New York State (USA-NY-22). Phylogenetic analysis of the capsid region further supported this genetic link (Fig. 2).

**Fig. 3:**
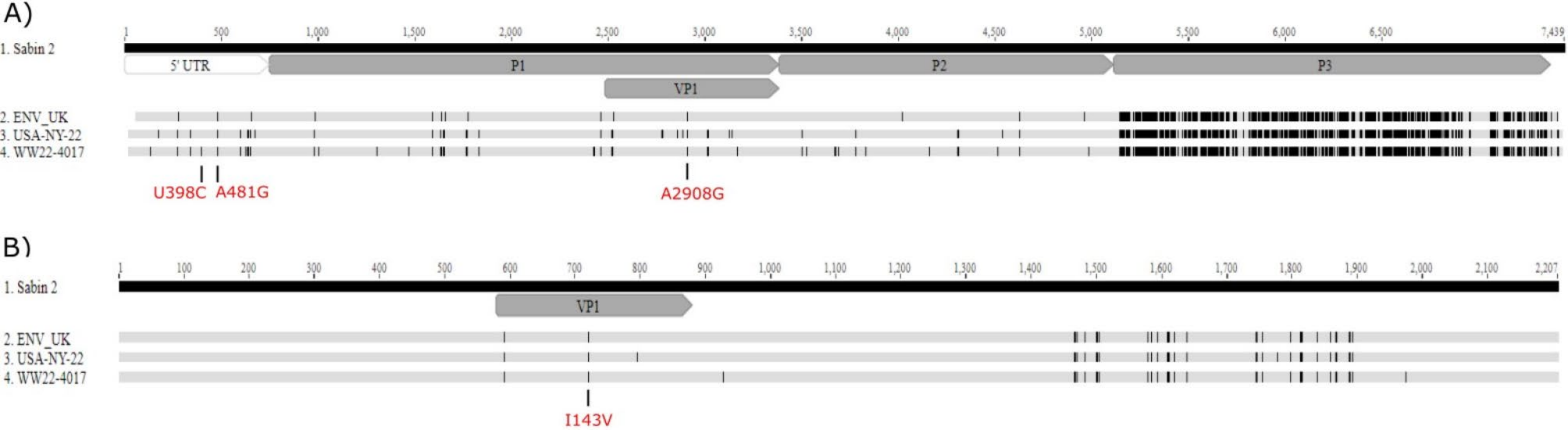
Mutation profile for the WW22-4017 poliovirus isolate. (**A**) Nucleotide and (**B**) amino acid mutation profiles of WW22-4017 (PQ641085) as compared to Sabin 2 (AY184220.1), USA-NY-22 (OP265178.1) and London UK environmental sample (OP410366.1). Mutations resulting in enhanced neurovirulence are depicted. UTR = untranslated region; P1 = polyprotein 1; VP1 = viral protein 1; P2 = polyprotein 2; P3 = polyprotein 3; WW = wastewater.

Whole genome sequence data of our culture isolate provided a unique opportunity to investigate the sequence profile of the VDPV2 detected in Canadian wastewater. We noted several nucleotide differences within the genome, some of which translated to non-synonymous substitutions when compared to the parental Sabin 2 strain (Fig. 3). Most notable substitutions were within the 3 main Sabin 2 attenuation sites. Specifically, nucleotides U398C and A481G within the 5’ UTR and A2908G (corresponds to amino acid I143V) within the VP1 capsid protein. These three mutations are known to enhance neurovirulence^25,26^ supporting viral reversion from the vaccine strain into a more neurovirulent wild-type form. Despite the lack of recombination analysis we did notice high homology within the P3 region between our viral isolate and the VDPV2 detections in London environmental samples in 2022^7^ and the New York paralytic case (Fig. 3), suggesting that a possible recombination event occurred under the same mechanism.

**Fig. 4:**
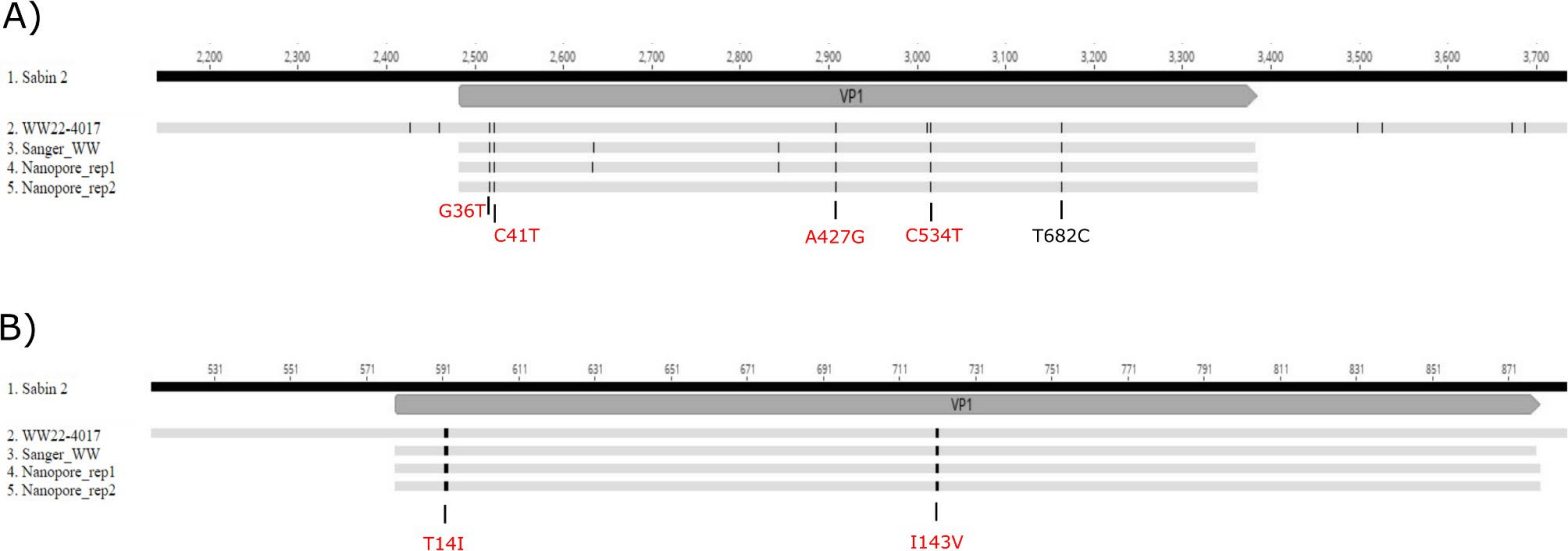
Mutation profile of the VP1 region sequenced by Nanopore and Sanger directly from the wastewater specimen WW22-4017. (**A**) Nucleotide and (**B**) amino acid differences as compared to Sabin 2 (AY184220.1) are depicted. Whole genome sequence data of WW22-4017 poliovirus isolate (PQ641085) is included for comparative purposes. Differences highlighted in red are the common ones shared with USA-NY-22. Sanger_WW (PQ641087), Nanopore_rep1 (PQ641089) and Nanopore_rep2 (PQ641090). WW = wastewater.

As part of this pilot study, we evaluated our direct detection method to determine whether it was sensitive to detect and sequence confirm the presence of the VDPV2 directly from wastewater. The same wastewater sample from which we obtained a culture isolate was used to PCR amplify the VP1 poliovirus region. Both Nanopore and Sanger sequencing of the VP1 PCR amplicon products of WW22-4017 confirmed the presence of VDPV2, noting 5–7 nucleotide differences from the Sabin 2 strain. We noted minor variations in sequencing results between the replicates and sequencing approaches. However, all observed variations were synonymous mutations (Fig. 4). Sequencing depth for all Nanopore sequencing runs ranged between ~ 295 000 to 330 000, indicating more than sufficient depth of coverage for the VP1 region to accurately generate a consensus (cut-off set at 50 reads). In addition to sequencing poliovirus, we did notice a second amplicon of ~ 1300 bp in length only in specimen WW22-4017 (Supplemental Figure S3). This amplicon accounted for ~ 47% of reads that were unmapped to poliovirus or other non-polio enteroviruses in the sample. Further investigation into the unmapped reads identified that 92.5% of these reads mapped to a region within one of 3 *Acidovorax sp.* bacterial genomes (Supplemental Figure S3) that have been previously found in urban sewage systems^27^. Regardless of the sequencing method used, the consensus VP1 region shared in common 4 nucleotide substitutions with USA-NY-22 as well as an additional substitution (T682C) that was present only in our sequence data. Our data supports the presence of poliovirus mixtures and/or variants of the same serotypes within wastewater samples, a phenomenon that has been well-documented for complex specimens such as environmental samples^7,23,28^.

Our direct detection method was sensitive enough to identify the presence of VDPV2 directly (culture-free) from the wastewater specimen. We therefore initiated a retrospective investigation into the potential presence of poliovirus using repurposed, historical COVID-19 wastewater samples collected from the north and south interceptor pipes between May-December, 2022. We also initiated prospective sampling of the north and south interceptor pipes between January 2023 until September 2024 to confirm that the region remained poliovirus-free. As of June 2024, only two additional samples that were collected from the north interceptor pipe on August 20th and 27th, 2022 tested positive for poliovirus using real-time PCR (Fig. 1 and Supplemental Table S1). Culture isolation of these two samples (re-purposed COVID-19 specimens) was attempted but were unsuccessful. Concurrently, PCR amplification directly from the wastewater specimen followed by sequencing of the VP1 region using Nanopore and Sanger resulted in successful sequencing of 1 of the samples tested (August 27th, 2022 collection). Sequence analysis of this sample confirmed the presence of 9 nucleotide substitutions within the VP1 region when compared to the Sabin 2 strain, verifying the presence of a VDPV2 (WW22-3857). Closer evaluation revealed that sample WW22-3857 also contained the same 4 nucleotide substitutions shared between specimen WW22-4017 and USA-NY-22 (Supplemental Table S4). Pair-wise analysis showed 99.2% identity to other environmental VDPV2 sequences detected in the UK and 98.8% sequence identity to the VDPV2 paralytic case detected in Rockland County, USA. Phylogenetic analysis revealed this sample to be genetically linked to the cVDPV2 sequence in New York (USA-NY-22) (Supplemental Figure S1). Translation of the VP1 region uncovered a total of 3 amino acid changes from Sabin 2 with I143V being the most notable.

## **Discussion**

This is the first study that detected and sequence verified the presence of vaccine-derived poliovirus type 2 (VDPV2) from Canadian wastewater. Samples collected on August 27th and 30th, 2022 in Montréal, Québec, were found to contain VDPV2 that were genetically linked to the New York paralytic case. Samples analyzed by both viral isolation and direct detection showed the same results, supporting utility of direct detection for wastewater surveillance. Additional surveillance activities did not detect poliovirus in either retrospective (May-December, 2022) or prospective samples (February 2023-June 2024), supporting the observation of a sporadic and brief period when poliovirus shedding occurred. No clinical case of poliomyelitis was reported in Canada via CAFPSS. We therefore suspect that the observed poliovirus detections in Canadian wastewater were travel-related, sporadic detections which did not result in community spread.

Whole genome sequencing of the clinical isolate revealed the presence of several nucleotide substitutions within vaccine attenuation sites that have a propensity to revert^25,26^. Specifically, two nucleotide substitutions within the 5’ UTR (U398C and A481G) as well as amino acid I143V change within the VP1 capsid protein. These 3 gatekeeper mutations are known to occur as the Sabin 2 vaccine regains neurovirulence properties^25,26^. Of note, the USA-NY-22 isolate as well as WW22-3857 specimen (collected 3 days prior to WW22-4017) did not contain the 5’ UTR U398C substitution that was found in specimen WW22-4017. It was recently identified that this gatekeeper mutation accumulates in vaccine-derived poliovirus genomes at ~ 75% frequency 4 weeks post-vaccination while the other two mutations have a slightly different timeline for reversion^29^. We therefore suspect that these two VDPV2 detections represent shedding events from different individuals that traveled to Canada from communities in the USA with circulating poliovirus. Whole genome sequencing of the isolated virus provided an opportunity to uncover the presence of a highly homologous P3 region to the one described in the UK London samples^7^ and also observed in the New York paralytic case. The UK London samples were found to have recombined with an enterovirus type C donor and we speculate that this P3 homology further strengthens the genetic relatedness among the VDPV2 detections in Canada, USA and UK. Recombination of poliovirus with another enterovirus type C is not a unique event and has been previously observed and documented for VDPV2^30^. The recombination event we observed resulted in several amino acid mutations within the P3 region. We noted 2 synonymous mutations within the 3C and 3D regions that were present in the UK, USA and our isolate (U5811A and U6693A). These mutations were found to naturally accumulate in individuals shedding the virus post-vaccination, suggesting a potential evolutionary advantage^29^. However, the effects of this recombination and observed amino acid change on viral fitness and neurovirulence remain to be determined. Of note, we did not observe any amino acid changes correlating with known antigenicity sites or antibody-neutralizing domains^13,26,31^. Overall, performing whole genome sequencing of poliovirus provides the highest resolution for obtaining accurate genetic linkage information, and allows for assessment of known neurovirulence traits.

One major consideration in establishing a functional targeted wastewater surveillance system in Canada is the necessary resources required to sustain such activities in response to an outbreak. Performing viral isolation through culture of all targeted wastewater sites can quickly overwhelm the laboratory resources needed for clinical surveillance^24,32^. As such, we were interested in evaluating a poliovirus direct detection method for wastewater application. Although direct detection is not currently the gold standard method for poliovirus surveillance, it is being investigated as an alternative^23^. In this study, we found that our direct detection method was successful at accurately identifying the presence of VDPV2 within the same wastewater sample from which we cultured and isolated the virus. The amount of poliovirus material in the tested sample was low because the PanPV real-time PCR assay had an indeterminate signal (37–40 Ct) and only 1 of 5 L20B culture flasks was positive. This weak poliovirus detection further corroborates that a travel-related shedding event contributed to the detection of poliovirus from this catchment rather than local transmission. Even with such a weak poliovirus signal, all Nanopore sequencing replicates generated reliable consensus sequences for this sample using direct detection. Our study adds additional support that direct detection of poliovirus from wastewater samples is possible using Nanopore sequencing^23^ and would greatly simplify and expedite environmental surveillance activities during an outbreak response. The extensive biocontainment requirements to perform viral culturing are not mandatory for the direct detection method. Furthermore, scalability to accommodate the timely processing of large batches of specimens can be easily attainable with the direct detection method. Additionally, culturing virus from complex specimens such as wastewater samples can lead to a bias in viral diversity detected in the sample because some of the viruses may outcompete low fitness variants in culture^33^. Lastly, direct detection can sequence confirm the presence of poliovirus within 3 days of sample receipt, much faster than the current gold standard culture method which takes at least twice as long to confirm the presence of poliovirus^23,34^. The fast turn-around time for the direct detection method would significantly expedite a public health response and allow for rapid implementation of outbreak control measures, directly translating into limiting poliomyelitis cases^19,35^. Overall, the time required to obtain a result from cell culture can have important consequences for the public health response because poliovirus can infect and shed from asymptomatic vaccinated and unvaccinated individuals^36^. If allowed to circulate undetected, the virus will have ample opportunity to evolve and regain neurovirulence^13^. It is therefore important to monitor for potential silent transmission using the most sensitive and least biased tools available.

One main limitation of this study was the infrequent sampling of the targeted sites. We were unfortunately unable to sample more frequently due to inclement weather conditions. Although all of the utility holes sampled do terminate into either the north or south interceptors, the amount of poliovirus may be too dilute to be reliably detected in the specimens collected from these interceptors. Furthermore, the north and south interceptors represented catchment area that were ~ 3 times larger than recommended by WHO, potentially affecting the sensitivity of detecting poliovirus from these sites^37^. As such, data presented here may not be representative of the potential extent of the poliovirus signal within the targeted communities. Furthermore, negative results presented in this study should be interpreted with caution because we did not perform extensive verifications for assay sensitivity and limits of detection. This applies to our modified, two-step real-time PCR assay as well as our poliovirus VP1 PCR used as input for Nanopore and Sanger sequencing.

Overall, this study highlights the utility of using a targeted poliovirus environmental surveillance approach to detect potential poliovirus importation within monitored communities. We were actively searching for potential poliovirus importation and/or silent community transmission as was observed in New York State^4^. Wastewater samples were specifically chosen that represented at-risk communities with known travel ties to communities in New York State with active poliovirus transmission^38^. Detection of poliovirus signal in the catchment servicing these communities does not necessarily mean that it was a member of this community that was shedding poliovirus. The sporadic detection of VDPV2 in Canadian wastewater samples collected 3 days apart indicated that our approach was sensitive to detect and sequence verify the presence of poliovirus in wastewater targeting catchment areas servicing between 18 000–1 000 000 individuals. Sequence data from the cultured viral isolate and direct detection methods genetically linked our poliovirus positive samples to poliovirus that circulated in New York State in 2022. Through this pilot study, we have shown that Canada has the capability of performing targeted environmental surveillance to enhance national poliovirus surveillance activities in response to a poliovirus outbreak or event. This work further supports the utility of poliovirus wastewater surveillance as an early warning system prior to the detection of clinical cases^39^. Monitoring wastewater for silent viral circulation is also important to confirm polio-free status within affected communities post-detection. Since silent viral spread accommodates increased viral fitness and reversion to a neurovirulent form that is associated with poliomyelitis, it becomes essential to utilize all tools available to confirm the interruption of viral transmission within communities where poliovirus is circulating. This capability becomes increasingly vital as the world nears poliovirus eradication slated for 2026^40^.

## **Materials and methods**

### Wastewater sample collection and processing

Wastewater samples from the north and south interceptors servicing Montréal’s Jean-R. Marcotte wastewater treatment plant were collected in support of the Public Health Agency of Canada, Statistics Canada Wastewater Joint Collection initiative. An additional 8 targeted sewage sites were directly sampled from utility holes within the City of Montréal. The populations serviced and weeks of collection for all sites are provided in Fig. 1. For all sites, wastewater was collected from raw influent as consecutive 24-hour composite samples with a final volume of 450–500 mL. Collections of the majority of samples occurred approximately every two weeks. Collections from targeted utility holes were less frequent due to inclement weather conditions.

Between 15 mL (re-purposed COVID-19 samples) or 50 mL of wastewater was clarified by adding 0.01% Tween-80 (Sigma-Aldrich, Oakville, Canada), vortexed for 20 s and centrifuged for 22 min at 4 198 x g. The 50 mL of clarified wastewater was concentrated to approximately 5 mL using a Jumbosep™ centrifugal device with a 10 kDa filter (Pall Corporation, Port Washington, USA) and centrifugation (4 °C for 40 min at 3 000 x g). The retentate or the 15 mL clarified wastewater sample was transferred into an Amicon^®^ Ultra-15 centrifugal device with a 10 kDa molecular weight cut-off (Millipore-Sigma, Burlington, USA) and centrifuged (4 °C for 35 min at 4 198 x g) to further concentrate the sample to approximately 200 µL for RNA extraction.

### RNA extraction of wastewater samples

RNA extraction was performed using the MagNA Pure 96 (MP96) system and the DNA and Viral NA Large Volume Kit (Roche, Mississauga, Canada) according to manufacturer’s recommendation. A total of 200 µL of concentrated wastewater sample was used as input plus 2 µL of carrier RNA added directly to 800 µL of Roche External Lysis buffer as a pre-lysis inactivation step. The concentrate, carrier RNA, and lysis buffer were incubated at room temperature for 10 min. RNA was extracted using the Viral NA Plasma ext lys LV program on the system (Roche). A total of 100 µL of RNA was eluted, separated into 3 aliquots and frozen at -25 degrees Celsius until processing.

### Real-time PCR

Two different real-time PCR methods were compared to evaluate their utility in detecting poliovirus and non-polio enteroviruses from wastewater samples. Both methods used the same primers and probes present in the Poliovirus rRT-PCR ITD 5.2 kit provided by the International Reagent Resource (IRR) and Centre for Disease Control (Atlanta, GA, USA). PanEV-specific primers and probes detect numerous enteroviruses including poliovirus while PanPV-specific primers and probes only detects poliovirus serotypes^41,42^. We used the PanEV real-time PCR assay as a process control to identify wastewater sample suitability for poliovirus testing due to the fact that enteroviruses are highly prevalent in humans and readily detected in wastewater samples^43^. All samples were processed using the CFX96 touch real-time PCR detection system (BioRad, Mississauga, Canada). Interpretation of real-time PCR results: Ct < 37 is positive signal while Ct 37–40 is indeterminate.

The one-step real-time PCR method using the qScript XLT one-step RT-qPCR ToughMix (Quanta Biosciences, Beverly, USA) was followed as previously described without modifications^41,42^. The modified assay consisted of a two-step real-time PCR assay. Briefly, 5 µL of RNA was converted to cDNA using 5 µL of SuperScript IV VILO master mix kit containing random and oligo(dT) primers (Thermo Fisher Scientific, Mississuaga, Canada) following manufacturer’s recommendation. Samples were incubated at 25 °C for 10 min, 55 °C for 20 min, and 85 °C for 5 min using the GeneAmp PCR System 9700 (Thermo Fisher Scientific). A total of 5 µL of cDNA was mixed with 1 µL primer/probe mix (Quadruplex EV + Sabin or PanPV) from the Poliovirus rRT-PCR ITD 5.2 kit, 10 µL PrimeTime Gene Expression Master Mix (Integrated DNA Technologies, IA, USA) and 4 µL water (Thermo Fisher Scientific) which was then amplified using the CFX96 touch real-time PCR detection system as previously described^41,42^.

### Sequencing of VP1

All wastewater samples were processed for VP1 PCR amplification and sequencing regardless of the PanEV real-time PCR assay results. Briefly, 10 µL of RNA was converted to cDNA using 10 µL of SuperScript IV VILO master mix kit containing random and oligo(dT) primers (Thermo Fisher Scientific) as per manufacturer’s recommendation. Samples were incubated at 25 °C for 10 min, 55 °C for 20 min, and 85 °C for 5 min using the GeneAmp PCR System 9700 (Thermo Fisher Scientific). PCR amplification of the poliovirus VP1 region was performed using a pool of Sabin 1, 2, 3 primers (10 µM) as previously described^24^. A total of 5 µL of cDNA was amplified using the Platinum SuperFi II PCR Master Mix kit (Thermo Fisher Scientific) according to the manufacturer’s recommendations for a 50 µL reaction. Thermocycler conditions were: 98 °C for 3 min, 45 cycles of 98 °C for 15 s, 60 °C for 30 s and 72 °C for 30 s, followed by final extension at 72 °C for 5 min using the Proflex PCR System (Thermo Fisher Scientific). PCR amplicons were visualized using QIAxcel Connect (Qiagen, Toronto, Canada). PCR products were purified using AMPure XP beads (Beckman Coulter, IN, USA) and 80% ethanol as described^44^. Cleaned PCR products were resuspended in 50 µl nuclease-free water and sample was split and processed on Nanopore and Sanger sequencing platforms.

Nanopore sequencing of the PCR products was performed using the Ligation Sequencing Kit (SQK-LSK109) and Native Barcoding Kit expansion pack (SQK-NBD196) following the manufacturer’s protocol (Oxford Nanopore Technologies, Oxford, UK) with a few modifications. Briefly, a 200 ng input DNA was used for the end-prep reaction followed by 20 °C for 10 min and 65 °C for 10 min of incubation. During native barcode ligation, the reagent volume was modified to 3 µL nuclease-free water, 1.3 µL Native barcode, 1.1 µL End-prepped DNA, and 5.0 µL Blunt/TA Ligase master mix (New England Biolabs, Whitby, Canada). The final library was loaded onto the flow cell (FLO-MIN106D) version R9.4.1 and sequenced on the GridION for 24 h enabling live base-calling using a high-accuracy model with software MinKNOW version 22.10.7 and Guppy version 6.3.9.

Sanger sequencing of the VP1 PCR amplicons was performed using the ABI Prism Big Dye Terminator Cycle Sequencing kit (Applied Biosystems, CA, USA) and the 3730XL DNA Analyzer (Applied Biosystems, CA, USA). Sequences were analyzed using the Sequencher program (GenCodes, MI, USA).

### Viral isolation and RNA extraction

Either 50 mL (re-purposed COVID-19 samples) or 150 mL of clarified wastewater was filtered through a 0.45 µM filter. Filtrate was concentrated 50 mL at a time using the Jumbosep™ centrifugal device with a 10 kDa filter as described above. Each sample was then concentrated further to a final volume of 4–6 mL for culture inoculation. Subsequently, virus isolation was performed following the WHO gold standard protocol^15^. Briefly, 0.5 mL of sample concentrate was inoculated into 5 separate transgenic murine cells expressing the CD155 human poliovirus receptor (L20B) and 3 separate human rabdosarcoma (RD) 25cm^2^ flasks. The LB20 (GR-924) and RD cells (GR-925) were obtained through the International Reagent Resource, Division of Viral Diseases, Centers for Disease Control and Prevention, Atlanta, GA, USA, in collaboration with the World Health Organization and the Global Polio Eradication Initiative. Cultures were monitored for cytopathic effect for 5 days. A total of 200 µL of cell culture medium was collected for RNA extraction from cultures exhibiting cytopathic effect. RNA was extracted and eluted in 50 µL using the KingFisher Flex Extraction System with the MagMax Viral/Pathogen kit (Applied Biosystems, ON, CA)^15^.

### Whole genome sequencing of culture isolate

RNA extracts from the cultured viral isolate was transcribed to cDNA using a Maxima H Minus Double-Stranded cDNA Synthesis Kit using random hexamers (Thermo Fisher Scientific) as per the manufacturer’s protocol. The double-stranded cDNA products were purified using 1x Ampure XP beads following the manufacturer’s recommendations. Sequencing library preparation was performed by using the Illumina DNA Prep kit following the manufacturer’s protocol. A total of 200 ng of purified cDNA was used as initial input. Libraries were diluted to a final concentration of 1 nM and sequenced on the iSeq 100 (2 × 150 bp, 300-cycle) (Illumina, CA, USA).

### Bioinformatics analysis

Nanopore generated sequence data were first dehosted by mapping the reads to the human hg38 reference genome using minimap2 (version 2.17-r941). The unmapped, non-human reads were collected and converted back to fastq files using samtools (version 1.14) and analyzed using the Poliovirus Investigation Resource Automating Nanopore Haplotype Analysis (PIRANHA version 1.0.6) bioinformatics pipeline developed by the Rambaut group at the University of Edinburgh as part of the Poliovirus Sequencing Consortium^45^. Sequenced reads were filtered to contain 300–2 000 nucleotides in length and these were mapped against the PIRANHA enterovirus VP1 reference panel. Mapped reads were parsed to the closest reference virus and consensus sequences were generated for references that composed at least 1% of all sample reads with positions requiring a minimum read depth of 50. Generated consensus sequences were 903 bp in length. Unmapped reads for sample WW22-4017 were further filtered based on size using Trim Reads (version 3.0) in CLC Genomics Workbench (version 24.0.1) and only reads containing > 1 000 nucleotides in length were further analyzed. Random sampling of 10 reads followed by blastn^46^ identified 3 *Acidovorax sp.* bacterial genomes as the top hits. Default setting of the Map Long Reads to Reference tool (version 1.2) was used to map the unmapped reads against the reference file containing the 3 bacterial genomes (CP021648.1, CP152342.1, CP033069.1).

Illumina generated sequence data were first dehosted by mapping the reads to the human hg38 reference genome using bowtie2 (version 2.4.5). The unmapped, non-human reads were collected and analyzed using the CLC Genomics Workbench (version 24.0.1). The quality of the sequencing reads was checked using QC for Sequencing Reads (version 0.2). Reads were filtered based on size using Trim Reads (version 3.0) and only reads containing 100–200 nucleotides in length were subsequently mapped to the reference sequence (OP265178.1) using the default setting in Map Reads to Reference (version 1.9). We chose to use the OP265178.1 reference to generate a consensus because it was the closest reference genome to our sample. Consensus sequence (7 408 bp in length) was extracted having a minimum read depth of 20 and visualized using Aliview (version 1.28).

Pair-wise analysis of consensus sequences generated via Nanopore, Illumina or Sanger sequencing was performed using the MUSCLE alignment in Geneious version 10.2.6. Reference sequences for this analysis included Sabin 2 (AY184220.1), New York clinical isolate (OP265178.1) and one of the UK environmental specimens closely related to the New York isolate based on our phylogenetic analysis (OP410366).

### Phylogenetic analysis

The poliovirus VP1-VP4 region was aligned using MUSCLE (version 3.8.425) and visualized in Aliview (version 1.28). The maximum likelihood phylogeny was constructed using PhyML 3.0^47^ and the model (GTR + G) was selected using Smart Model Selection in PhyML with aLRT SH-like branch supports^48^. Tree was rooted with Sabin 2 and image edited in iTOL (version 6.9.1).

The nucleotide alignment of poliovirus VP1 sequences was created using whole genome sequence data and trimmed to generate a complete VP1 alignment by removing 1-2 362 nucleotides from the 5’ end and 3 266–7 283 nucleotides from the 3’ ends using Aliview. The maximum likelihood phylogeny was constructed using PhyML 3.0 and the K80 + G model was selected using SMS with aLRT SH-like branch supports. Tree was rooted with Sabin 2 and image edited in iTOL.

## Electronic supplementary material

Below is the link to the electronic supplementary material.

Supplementary Material 1.

Supplementary Material 2.

Supplementary Material 3.

## Data Availability

Sequence data can be found within the BioProject ID (PRJNA1186727). Demultiplexed and dehosted datasets have been provided. All generated FASTA sequences are deposited in NCBI under PQ641085-PQ641090.
