# Supplementary material for: Sporadic detection of vaccine-derived poliovirus type 2 using next-generation sequencing in Canadian wastewater in August of 2022
Source: Sci Rep. 2025 Apr 15;15:12913. doi: 10.1038/s41598-025-92912-x (PMC12000465; doi:10.1038/s41598-025-92912-x)
Supplement: Supplementary file 3 — Supplementary Material 3. [file 41598_2025_92912_MOESM3_ESM.docx]

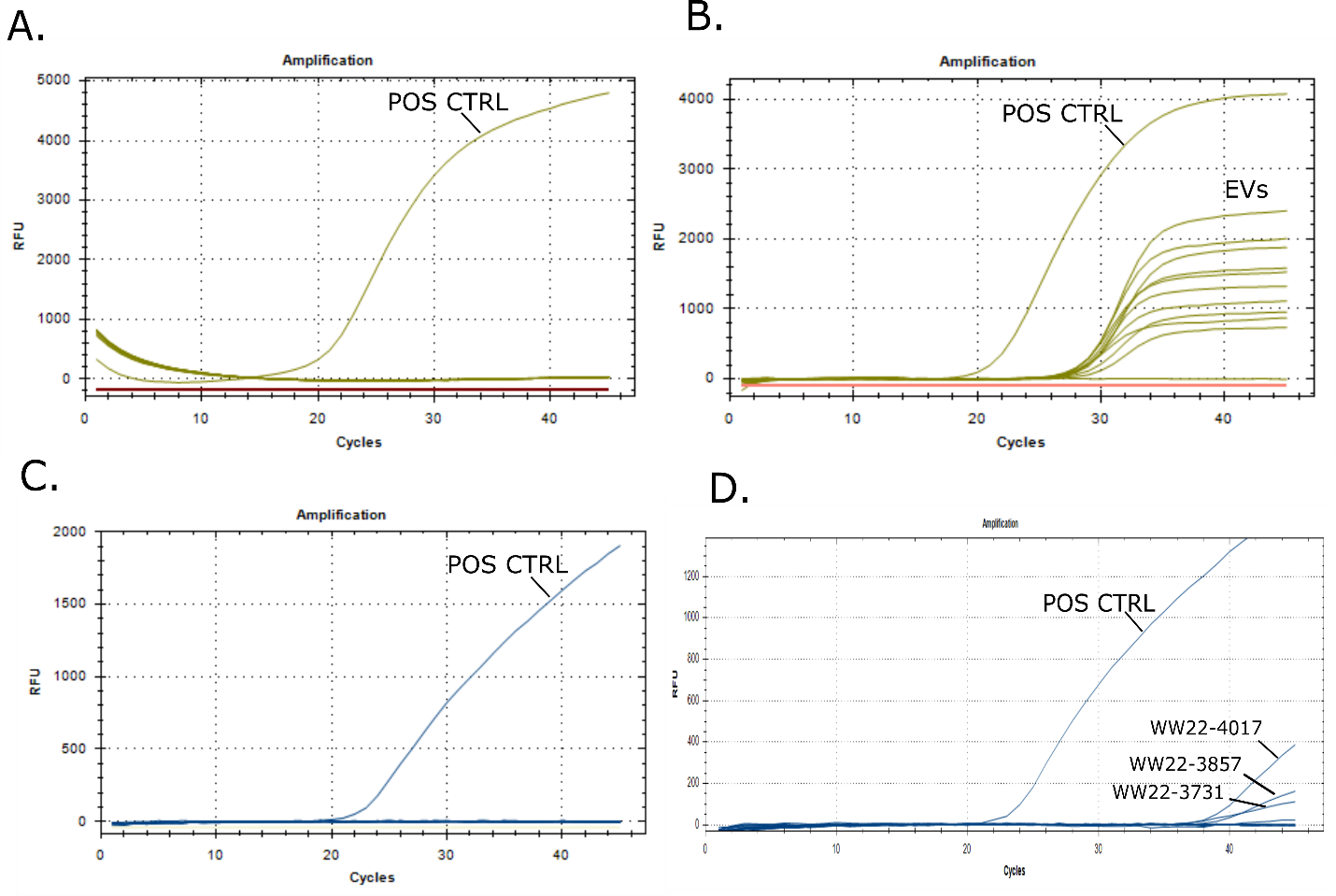


**Supplemental Figure S1. Real-time PCR amplification curves for the PanEV and PanPV assays used in this study.** All assays were run on wastewater specimens and results provided represent both unmodified (A and C) and modified (B and D) assay conditions. Assay comparisons consists of 10 wastewater samples for the PanEV (A and B) and 16 wastewater samples for the PanPV (C and D) assay. Processing controls included no template control (NTC) and positive control (POS CTRL) provided in Poliovirus rRT-PCR ITD 5.2 Kit. For these assays the wastewater samples and their Ct values are depicted in Supplemental Table S1. Poliovirus was detected in WW22-4017, WW22-3857 and WW22-3731 wastewater samples as indicated. EVs = enteroviruses.

**
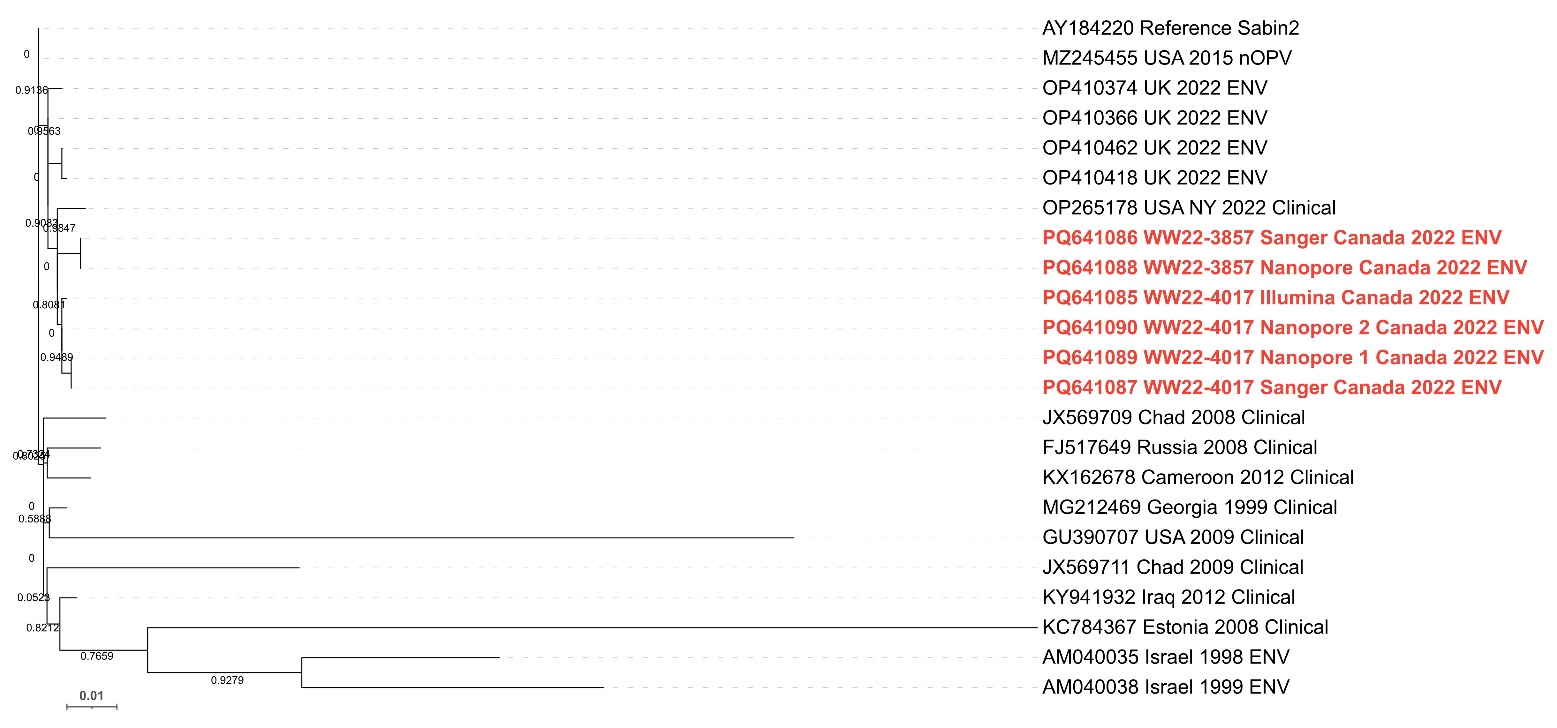
**

**Supplemental Figure S2.** **Phylogenetic analysis of poliovirus VP1 nucleotide sequences.** Select sequences were obtained from NCBI GenBank database. Red text highlights the poliovirus type 2 sequences obtained from Canadian wastewater in 2022 as part of this study. All Canadian sequences generated by either Sanger or Nanopore represent data directly obtained from wastewater samples. The WW22-4017 Illumina Canada 2022 ENV sequence was generated from the cultured viral isolate. The tree was rooted with a Sabin 2 reference. SH-like branch support values are indicated at the nodes and the maximum-likelihood scale bar indicates average residue substitution per site. ENV = environmental.


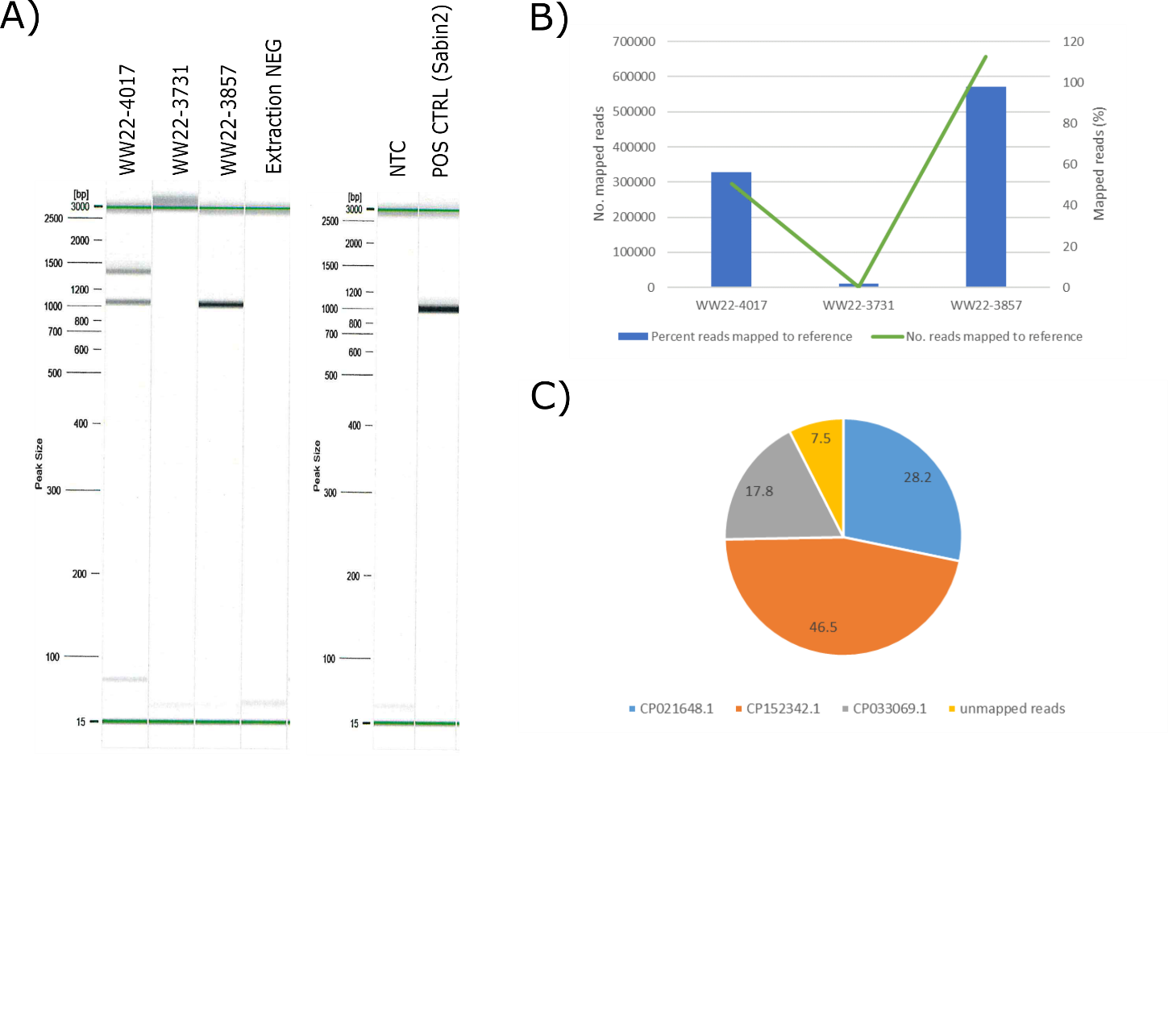


**Supplemental Figure S3. PCR amplification of the poliovirus VP1 region directly from wastewater samples.** A) PCR products visualizes on the QIAxel Connect System for samples determined as either positive or indeterminate for poliovirus signal using the modified PanPV real-time PCR assay. Processing controls included extraction negative (NEG), no template control (NTC) and Sabin2 extracted RNA from culture as positive control (POS CTRL). B) Bioinformatic analysis results of the Nanopore sequence data for WW22-4017, WW22-3731 and WW22-3857 wastewater specimens depicting the number of reads that mapped to the poliovirus Sabin2 (AY184220) reference. C) Percent of the unmapped reads fraction (177 858 reads that were unmapped and >1 000 bp in length) in samples WW22-4017 that mapped (164 527 reads = 92.5%) to one of the 3 *Acidovorax* *sp.* delineated by the specific NCBI accession number.

| **Supplemental Table S3.** Nucleotide substitutions observed within the VP1 sequence poliovirus culture isolated from specimen WW22-4017. | | | |
| --- | --- | --- | --- |
|  |  |  |  |
| **Nucleotide Position VP1** | **Reference** | **Sanger** | **Illumina** |
| 36 | G | **T** | **T** |
| 41 | C | **T** | **T** |
| 427 | A | **G** | **G** |
| 531 | G | A | A |
| 534 | C | **T** | **T** |
| 682 | T | C | C |
| Total mutations |  | 6 | 6 |
| NGS read depth |  |  | 27 472 |
|  |  |  |  |
| Bolded nucleotide indicates common nucleotide substitution shared with the USA-NY-22 (OP265178.1) isolate. | | | |
|  | | | |

| **Supplemental Table S4.** Nucleotide substitutions observed within the poliovirus VP1 region directly sequenced from wastewater sample WW22-3857. | | | | |
| --- | --- | --- | --- | --- |
|  |  |  |  |  |
| **Nucleotide Position VP1** | **Reference** | **Sanger** | **Nanopore** |  |
| 9 | T | C | C |  |
| 36 | G | **T** | **T** |  |
| 41 | C | **T** | **T** |  |
| 79 | C | T | T |  |
| 106 | G | T | T |  |
| 201 | C | T | T |  |
| 219 | A | G | G |  |
| 427 | A | **G** | **G** |  |
| 534 | C | **T** | **T** |  |
| Total mutations |  | 9 | 9 |  |
| NGS read depth |  |  | 656 734 |  |
|  |  |  |  |  |
| Bolded nucleotide indicates common nucleotide substitution shared with the USA-NY-22 (OP265178.1) isolate. | | | |  |
